# Immune Response to Mycobacterial Infection: Lessons from Flow Cytometry

**DOI:** 10.1155/2013/464039

**Published:** 2013-11-27

**Authors:** Nikoletta Rovina, Marios Panagiotou, Konstantinos Pontikis, Magdalini Kyriakopoulou, Nikolaos G. Koulouris, Antonia Koutsoukou

**Affiliations:** ^1^Intensive Care Unit, 1st Department of Respiratory Medicine, Medical School, National and Kapodistrian University of Athens and “Sotiria” Chest Disease Hospital, 152 Mesogeion Avenue, 11527 Athens, Greece; ^2^3rd Department of Pulmonology, Sismanoglio General Hospital, 15126 Athens, Greece; ^3^1st Department of Respiratory Medicine, Medical School, National and Kapodistrian University of Athens and “Sotiria” Chest Disease Hospital, 11527 Athens, Greece

## Abstract

Detecting and treating active and latent tuberculosis are pivotal elements for effective infection control; yet, due to their significant inherent limitations, the diagnostic means for these two stages of tuberculosis (TB) to date remain suboptimal. This paper reviews the current diagnostic tools for mycobacterial infection and focuses on the application of flow cytometry as a promising method for rapid and reliable diagnosis of mycobacterial infection as well as discrimination between active and latent TB: it summarizes diagnostic biomarkers distinguishing the two states of infection and also features of the distinct immune response against *Mycobacterium tuberculosis* (*Mtb*) at certain stages of infection as revealed by flow cytometry to date.

## 1. Introduction

Following diagnosis of mycobacterial infection, distinguishing active from latent tuberculosis is critical because the management of the patient diverges between the two stages; a targeted approach based on clear-cut diagnosis graces the patients with more efficient treatment avoiding their exposure to unnecessary and potentially harmful interventions.

Targeting latent tuberculosis (LTBI) is a widely acknowledged priority for tuberculosis control. LTBI affects a significant share of the world population today. In 2000, in the United States alone, an estimated 11,213,000 residents representing 4.2% of the civilian, noninstitutionalized population aged >1 year had LTBI; of these persons, only 25.5% had been diagnosed, and only 13.2% had been prescribed treatment [1, 2]. Attacking this vast reservoir of mycobacterial infection offers a unique opportunity for a vital strike against mycobacterial infection; treatment of LTBI can reduce the risk of development of disease by as much as 90 percent, thus benefiting the individual as well as the public health. Targeting LTBI becomes even more important nowadays due to the increasing frequency of patients susceptible to developing active TB. To this latter category belong the growing group of patients receiving immunosuppressive therapies such as corticosteroids or antitumor necrosis factor-alpha (TNF-*α*), transplant patients, and those with HIV infection all of whom are at increased risk of rapid progression of a recently acquired tuberculous infection and of reactivation of latent TB infection [3–6].

In the absence of gold standard, flow cytometry attempts to provide an efficient method of decoding the immune response to the *Mtb*, development of efficient immune-based interventions, and also rapid diagnosis of mycobacterial infection. Following a brief review of the currently available diagnostic options for mycobacterial infection, this article focuses on the available evidence evolving from flow cytometry to date that shed light on the distinct immunological pathways of active and latent TB and summarizes diagnostic biomarkers that enable discrimination between the two stages of infection.

## 2. Current Diagnostic Options

Traditionally, the diagnosis of active TB is based on epidemiological, clinical, and radiographic features and warrants microbiological or histopathological confirmation [2, 3]. This strategy poses a significant burden on the patient and the healthcare resources: it is slow and laborious, and also pending the results, the patient may be hospitalized and submitted to presumptive treatment accepting the chance that the screening may ultimately turn out to be negative [4–7].

For the identification of LTBI, two major tests evaluating T-cell-mediated immunity are commonly employed: the tuberculin skin test (TST) and the interferon gamma release assays (IGRAs). Positivity of either of these tests in the absence of clinical signs or symptoms for active TB establishes the diagnosis of LTBI [8–10].

IGRAs are currently supplanting TST for the diagnosis of LTBI due to their greater reliability [8–10]. Modern IGRAs are based on in vitro T-cell stimulation with the immunodominance and high specificity for *Mtb,* 6-kDa early secretory antigenic target (ESAT-6), and 10-kDa culture filtrate antigen (CFP-10) which are encoded in region of difference 1 of the mycobacterial genome. IGRAs assess the expression of interferon-*γ* (INF-*γ*) in the T-cells. To date, two commercially available IGRAs exist: the QuantiFERON-TB Gold In-Tube test (QFT-GIT) and T-SPOT. TB test (T-Spot). The QFT-GIT test measures the amount of INF-*γ* in the supernatant of a cell suspension, whereas the T-Spot determines the number of cells producing INF-*γ* with the use of an ELISpot assay. Due to their high sensitivity (pooled sensitivity 81% for QFT-GIT and 91% for T-spot) and specificity (pooled specificity 99% for QFT-GIT and 88% for T-spot in low-risk populations) for the diagnosis of active TB, these assays provide rapid supplementary tools for screening the disease [8–10]. Importantly, both ESAT-6 and CFP-10 are absent from BCG vaccine strains and most nontuberculous mycobacteria (NTM) and this allows IGRAS not to be confounded by these factors (Table 1).

Despite being generally advantageous over TST, IGRAs are subject to limitations. IGRAs use IFN-*γ* as the sole read-out marker of cellular immunity and provide only limited biological information that is clinically interpreted in a binary fashion, whereby a result indicates merely the presence or absence of infection [8]. Although the diagnostic sensitivity of both IGRAs seems to be higher than that of TST for the diagnosis of active TB, it is still not high enough to use these assays as a rule-out test for TB and the specificity of IGRAs to distinguish patients with active TB from LTBI is still inadequately low [9]. The diagnostic role of IGRAs in the early stages of infection is also less clear as contradictory results have been obtained with recently exposed subjects. Both TST and IGRAs lack specificity for predicting reactivation of tuberculosis. In the general immunocompetent population, only about 5% with a positive TST will progress from LTBI to disease in their lifetime. In an analysis of four studies conducted among household contacts of persons with active TB, the pooled sensitivity of IGRAs for predicting the development of active disease within several years after exposure was 80 to 90%, the specificity 56 to 83%, the positive predictive value (PPV) 4 to 8%, and the negative predictive value (NPV) 99 to 100% [11]. IGRAs are also unsuitable for monitoring treatment response or test of cure TB: although IFN-*γ*-secreting T-cells measured by IGRAs often decline at the population level in TB patients during treatment, this trend masks substantial interindividual variation with some patients showing no decline and others showing an increase [12]. Furthermore, the use of IGRAs for serial testing and thus their applicability in screening programs, such as those for health care workers, are complicated by the lack of data on optimum cut-offs for serial testing and unclear interpretation and prognosis of conversions and reversions [13].

Of note, similar to TST, the diagnostic performance of IGRAs is hampered by the presence of immune suppression [4–7, 14] due to their dependence on an intact immune system capable of mounting an efficient immune response to mycobacterial antigens. In a systematic review and meta-analysis of 5,736 HIV-infected individuals, the sensitivity of IGRAs for active TB was reduced to 61% for QFT-GIT and 72% for T-spot [15]. Due to immaturity of the immune system, IGRAs are also problematical in children in whom IGRAs have shown to have pooled sensitivity for diagnosing infection with *Mtb* being as low as 66% with high heterogeneity [16].

Last but not least, an increasingly acknowledged limitation of IGRAs in diagnosing *Mtb* infection is the measurement of a single functional parameter, namely, IFN-*γ*, which may not be the most effective marker of the presence of *Mtb* infection, especially when evaluated alone. Antigen-specific T-cells secrete a plethora of cytokines, and clearly other biomarkers might be useful in distinguishing between latent and active TB. In support of this fact, Harari et al. [17] demonstrated that single-positive TNF-*αMtb*-specific CD4^+^ T cells were increased in subjects with active disease and this parameter was the strongest predictor of diagnosis of active versus latent infection. Furthermore, Hughes et al. [18] demonstrated the enhanced sensitivity which can be achieved when using several different cytokines simultaneously. This underscores the notion that IFN-*γ* should not be used as a sole read-out for T-cell activation by TB antigens.

To conclude, TST and IGRA undoubtedly entail significant inherent limitations. Effective control of tuberculosis warrants the development of rapid and more reliable immunological test for mycobacterial infection.

## 3. Flow Cytometry

Flow cytometry is a powerful technique for the analysis of multiple parameters of individual cells within heterogeneous populations. It is used in a range of applications from immunophenotyping to ploidy analysis, cell counting, and gene expression analysis. The flow cytometer performs this analysis by passing thousands of cells per second through a laser beam and capturing the light that emerges from each cell when passing through it. The data gathered can be analyzed statistically by flow cytometry software to report cellular characteristics such as size, complexity, phenotype, and health [19].

In the context of tuberculosis, flow cytometry is currently still confined to the research field and, therefore, has limited impact on the clinical management of the patient. However, the number of studies using flow cytometry for exploring the immune response against TB has been increasing in recent years and promising evidence suggests that it may serve not only as useful research tool but also as a valuable clinical apparatus due to its advantages over the available immune-based tests to date. Flow cytometry not only defines the numbers of cells producing a given cytokine but also allows the phenotypic differentiation between antigen-specific lymphocyte subsets in various states of TB infection [20]. Importantly, the diagnostic performance of flow cytometry in TB is not hindered by the presence of immunosuppression such as HIV-infected patients who represent a big and challenging reservoir of the mycobacterial infection worldwide.

## 4. Immune Response to Mycobacterial Infection

Flow cytometry studies utilize the fact that the heaviest (in quantity, antigenicity and duration) the antigenic exposure of T-cells to TB antigens is, the more advanced the cellular differentiation will be reached. It is only through decrypting that increment of differentiation that meaningful conclusions about the immune response to *Mtb* can be drawn. For this purpose, similar to IGRAs, flow cytometry methodology includes boosting T-cell differentiation by antigenic stimulation through incubating biological samples with combinations of tuberculin purified protein derivative (PPD) and the *Mtb*-specific antigens ESAT-6 and CFP-10.

Upon infection *Mtb* primarily stimulates type 1 (Th1) cytokine reaction that is driven by the CD4^+^ T-cells. Key cytokines in this reaction are IFN-*γ* and TNF-*α* which synergize to activate microbicidal effector mechanisms in human macrophages. This is the base of the delayed-type hypersensitivity caused by *Mtb* antigens and this phenomenon has been used for more than a century to identify *Mtb*-infected subjects by the TST [21]. It has been postulated that quantification of the immune response could potentially serve as a discriminating test for the different stages of mycobacterial infection. Supportive of the hypothesis was a study of 62 patients showing that the amount of CD4^+^-specific response is correlated with antigen burden. Particularly, *Mtb*-specific CD69^+^IFN-*γ* ^+^ CD4^+^ T-cells were higher in patients with recent contacts of patients with active TB (RC-TB) compared to BCG-vaccinated healthy controls (BCG-HC) and health care workers presented a higher frequency of these cells with respect to BCG-HC subjects but not different from RC-TB subjects [22].

Albeit a minority, some patients infected with *Mtb* mount prominent Th2 responses [23] such as production of IL-10 in response to ESAT-6 [18]. It is of course difficult to be certain of the true and false-positive rates of *Mtb*-induced Th2 cytokines due to the limited size of these studies, but the results do indicate the diagnostic value of Th2 cytokines, which should be further considered by larger studies.

### 4.1. T-Cell Differentiation

Flow cytometry has allowed a simultaneous assessment of the phenotype and multiple effector functions of single T-cells; the delineation of T cells into distinct functional populations defines the quality of the response [24]. Following antigen exposure, CD4^+^ and CD8^+^ T-cells undergo differentiation through various stages. While the exact path of differentiation remains under exploration, a current mainstream hypothesis is that naïve T-cells (T_N_) progress through central memory (T_CM_) that secretes only IL-2, to effector memory (T_EM_) that secretes both IFN-*γ* and IL-2, and then to finally terminally differentiated effector memory (T_EMRA_) T-cells that secrete only IFN-*γ* [25]. These T-cell subsets correlate with antigen and pathogen load in vivo, as exemplified by studies of chronic viral infections and TB, and quantification of these key T cell subsets is a promising approach for diagnosis and monitoring of infectious diseases, as well as for identifying correlates of protective immunity and for measuring vaccine immunogenicity [12]. Flow cytometry attempts to draw associations of these phenotypic and functional signatures of CD4^+^ T-cells subpopulations with pathogen burden and antigen load and, ultimately, with different stages of mycobacterial infection. Evidence suggests that states of persistent low antigen load and long-term immune control such as treated TB, latent infection, and BCG vaccination are associated with an immunological profile dominated by dual IFN-*γ*/IL-2-secreting T_EM_ cells and IL-2-secreting T_CM_ cells; on the contrary, active TB is predominated by IFN-*γ*-only-secreting T_EMRA_ cells [12, 20, 26, 27]. Moreover, longitudinal tracking of the immune response during the substantial reductions in pathogen load induced by the induction phase of anti-TB treatment showed an increase in dual IFN-*γ*/IL-2-secreting T-cells after treatment suggesting that this shift could also serve as a biomarker of treatment response [27].

### 4.2. Polyfunctionality

Studies in the field of antiviral immunity have shown that the ability of antigen-specific T-cells to simultaneously produce a range of cytokines (i.e., polyfunctional T-cells) has been associated with superior functional capacity and has been correlated with the control of human chronic viral infections such as HIV and hepatitis C and other intracellular pathogens, such as *Leishmania* [28]. However, the existing data in human TB are currently inconclusive. In agreement with studies on antiviral immunity, studies of cytokine profiles by multiparametric flow cytometry demonstrated that *Mtb-specific *CD4^+^ T cell responses in LTBI were mostly (>70%) polyfunctional, producing IFN-*γ*, IL-2, and TNF-*α*. Simultaneously, the immunological response in active TB disease was predominated (>70% of CD4^+^ T-cells) by TNF-*α* only response thus demonstrating that TNF-*α*, and not the IFN-*γ* (which is the cytokine measured by IGRAs), clearly distinguishes between active and latent TB [25]. Elsewhere, bifunctional IFN-*γ* ^+^TNF-*α* ^+^ CD4^+^ T-cells are significantly associated with active TB compared to the LTBI group [21]. In contrast, a study in Gambian population demonstrated a significantly higher proportion of polyfunctional IFN-*γ* ^+^IL-2^+^TNF-*α* ^+^ CD4^+^ T-cells in subjects with active TB disease compared with TST^+^ (latently infected) or TST^*‒*^ (uninfected) household TB contacts [26]. The IFN-*γ*/IL-2/TNF-*α* triple expression by CD4^+^ T-cells was also detectable in as high as 85–90% of TB patients and as low as 10–15% of LTBI subjects in an Italian study [27]. These contradictory findings may be due to this particular cytokine profile not being protective against TB or due to the coexistence of other factors that militate against the efficacy of these cytokines on the effector cells that eliminate *Mtb* [26].

The principles of polyfunctionality also seem to apply to other T-cells populations as evidence suggests that there is a shift in the distribution pattern of cytokine expression of CD3^+^ T-cells from exclusively IL-2 or TNF-*α* expression seen in healthy controls towards an increased frequency of IFN-*γ*/IL-2 or IFN-*γ*/TNF-*α* coexpression in TB indicating an obviously disease-associated shift from early memory cellular subpopulations in controls to activated and type 1 biased T-cells in TB patients [29].

### 4.3. Suppression of the Immune Response

Active TB is characterized by a suppression of *Mtb*-specific T-cell response. Using CD4^+^ T-cell receptor tetramers, it has been shown that there is a relatively low level of *Mtb*-specific monocytes in the peripheral blood of patients with advanced and untreated TB compared to non-TB patients, healthy donors, and umbilical cords. This phenomenon is presumably related to the apoptosis or necrosis of antigen presenting cells such as macrophages due to live mycobacteria and their growth [19]. Elsewhere, a decreased proliferative response and production of IFN-*γ* affecting both CD4^+^ and CD8^+^ T cell subpopulations which occurred in active TB [30] and suppression of IL-17^+^ CD4^+^ T-cells were associated with active TB [31]. Furthermore, the naturally occurring CD4^+^CD25^+^FoxP3^+^ CD4^+^ regulatory T-cells (Tregs) have been shown to attenuate the ability of infected monocyte-derived and alveolar macrophages to restrict the mycobacterial growth in a concentration-dependent fashion. Higher levels of Tregs cells have been detected in BAL and peripheral blood of patients with TB compared to LTBI. Additionally, levels of Tregs cells gradually declined during successful therapy and persistence tightly correlated with multidrug-resistant TB. Collectively, these findings suggest that the induction of Tregs cells constitutes an important escape mechanism of *Mtb* [32–34].

### 4.4. Compartmentalization of the Immune Response

Evidence suggests the dominance of immune responses in the lung during mycobacterial infection. With regard to localization of the aforementioned Tregs, the alveolar lung compartment seems to be relatively enriched for Tregs compared to the blood and this is combined with a consonant compartmentalization of higher concentrations of IFN-*γ*, TNF-*α*, IL-17, and IL-22 in BAL versus blood in patients with active TB [32]. A study of 36 subjects examining the cytokine responses of CD4^+^ T-cells in BAL demonstrated strong IFN-*γ* and TNF-*α* responses among patients with pulmonary TB as opposed to healthy control subjects and in lung compared to peripheral blood [33]. Curiously, this marked difference in frequencies was also observed in patients with nonpulmonary TB and patients with disseminated TB in whom extrapulmonary complications dominated the clinical picture. This observation suggests that a lymphocyte recirculation pathway to the lung exists, which presumably reflects the fact that, even in non-pulmonary TB, the origin of postprimary disease was the lung. In any case, this finding has significant diagnostic implications for patients with nonpulmonary TB, for whom achieving a culture-based diagnosis may be both difficult and hazardous. Whether the *Mtb*-specific responses in the lung may be of greater importance than the immunological findings within peripheral blood remains to be proven by larger studies [33].

### 4.5. The Role of CD8^+^ T-Cells

The applicability of TST and IGRAs in CD4^+^-deprived patients due to HIV infection is significantly restricted. Decrypting the specific CD8^+^ T-cell responses to TB antigens may assist the development of reliable immunodiagnostic TB test for this important group of patients. A few studies looked into the role of CD8^+^ T-cells in active TB [33]. CD8^+^ T-cell response against the *Mtb*-specific antigens seems to be a signature of a recent exposure to *Mtb* as recent contacts of TB patients, independently of their response to QFT-GIT, are presenting a higher CD8^+^ T-cell response to the same antigens compared to the other study groups [22]. Evidence suggests that CD8^+^ T-cells are elevated in active TB and also may be primed for secretion of Th1 cytokines in patients with active TB [26, 35]. Whether the quality of Th1 responses generated by CD8^+^ T-cells is equivalent to that of CD4^+^ T-cells remains to be seen, but this may be a potential factor in disease [26].

## 5. Diagnostic Biomarkers of Active TB

Few studies attempted to establish new biomarker cut-offs for clinical tuberculosis based on measurable changes that *Mtb*-specific T-cells undergo as they differentiate in response to virulent mycobacteria [21].

In a study of 30 patients with TB at different stages of treatment and 31 control healthy individuals with no signs or symptoms of TB but various degrees of exposure (household contacts, health care workers in a lung hospital, medical students, and unexposed healthy blood donors), it was shown that the percentage of PDD-specific CD4^+^ T-cells lacking the surface receptor CD27 (a state associated with advanced differentiation due to increasing TB exposure) is highly discriminating between active and latent TB infections [36]. In this study, BCG-vaccinated controls had significantly fewer CD27^*‒*^CD4^+^ T-cells than patients with smear and/or culture positive pulmonary tuberculosis allowing for discrimination between these groups with high sensitivity and specificity whereas individuals with LTBI exhibited levels in-between. Moreover, the diagnostic value of CD27^*‒*^CD4^+^ T-cells was augmented if combined with secretion of IFN-*γ*. Specifically, the threshold of 49% of CD27-INF-*γ* ^+^ within CD4^+^ T-cells was identified as the best discriminating marker between patients and controls (sensitivity 100%, specificity 85.7%, PPV 100%, and NPV 94%). Those findings were successfully validated in a prospective blinded study of 31 subjects with smear and/or culture positive or smear and culture negative pulmonary TB. Patients with smear and/or culture positive TB could be readily discriminated not only from control patients but also from patients with smear and culture negative TB. Using a threshold above 48% in which cases would be diagnosed to have smear and/or culture positive TB, both sensitivity and specificity would be 100%; also, PPV and NPV would be 100% [36].

A study of analysis of cytokine profiles in 48 individuals with known diagnosis of *Mtb *infection in Switzerland showed a substantial increase in the proportion of single-positive *Mtb*-specific TNF-*α*-producing CD4^+^ T-cells in subjects with active TB [25]. In this study, Harari et al. analyzed the cytokine profile (interferon-g (IFN-g), tumor necrosis factor-a (TNF-a), and interleukin-2 (IL-2)) of *Mtb*-specific T cells by polychromatic flow cytometry. The proportion of single positive *Mtb*-specific TNF-*α*-producing CD4^+^ T-cells was the strongest predictor of diagnosis of active disease versus latent infection. In addition, a cut-off of 37.4% of single-positive TNF-*α* ^+^ CD4^+^ T-cells was calculated as the value allowing the best separation between latent infection and active disease (sensitivity of 100% and specificity of 96%). This cut-off was validated in a cohort of 101 subjects (72 participants from Switzerland and 42 from South Africa) with tuberculosis diagnosis unknown to the investigator. The sensitivity and specificity of the flow cytometry-based assay were 67% and 92%, respectively; the PPV was 80% and the NPV was 92.4%. Overall, the cytokine profile predicted the clinical diagnosis in 90% of cases. These results were also confirmed in five participants during untreated active tuberculosis disease and then after tuberculosis treatment [25].

A study of 24 patients with active untreated TB and 28 patients of nonactive state (including individuals with successfully treated TB, LTBI, and BCG vaccination or infection with NTM) revealed that frequencies of PPD-specific IFN-*γ* ^+^IL-2^+^ dual-positive T-cells below 56% were an accurate marker for active TB. This threshold had a specificity of 100% and sensitivity of 70% thus enabling effective discrimination from non-active states. This biomarker was successfully validated in 50 PPD-reactive healthy individuals without evidence for active TB (25 healthy individuals with LTBI infection and 25 subjects with BCG vaccination or NTM infection) [20].

Focusing on the lung as a primary site of infection, Breen et al. [37] demonstrated that a single sample obtained by simple sputum induction could be used for the prompt diagnosis of TB. In this prospective cohort study of 42 participants with spontaneous sputum smear-negative or sputum nonproducing adults who were under investigation for TB, flow cytometry was employed to measure PPD-specific IFN-*γ*-secreting CD4^+^ T-cells. In 27 subjects diagnosed with active TB, the median percentage of IFN-*γ*-secreting CD4^+^ T-cells was 2.77% versus 0% in 15 negative subjects for active infection. Using a cut-off for the median percentage of PPD-specific IFN-*γ* ^+^ CD4^+^ T-cells of 0.5%, the sensitivity and specificity of the immunoassay versus final diagnosis of active TB were 89% and 80%, respectively. Assay performance was unaffected by HIV status (with 38% of the subjects being HIV-positive), BCG-vaccination, or disease site. Combining this approach with traditional microbiological methods increased the diagnostic yield to 93% alongside acid-fast bacilli (AFB) smear and 96% alongside TB culture. Importantly, of the 15 subjects with active pulmonary TB, 13 were immunoassay positive whereas only 4 were AFB smear positive on a single induced sputum sample, showing that the former greatly increased the chance of prompt diagnosis of active, infectious pulmonary TB [37].

In a prospective-sectional blinded study of 28 individuals with suspected active TB, the positive cut-off of >0.01% *Mtb-specific *IFN-*γ*-producing CD4^+^ T-cells had a sensitivity of 94.1% for diagnosing active TB. The sensitivity of the flow cytometry compared favorably with that of QFT-GIT assay (88.2%), which was also employed in the study. Both assays showed poorer performance in terms of their specificities, which were 36.4% for the flow cytometry and 18% for QFT-GIT, when the strict criterion of a positive culture result for *Mtb* was used for comparison [35].

Elsewhere, TB patients had significantly higher frequencies of ESAT-6-specific CD69^+^ CD4^+^ T-cells secreting at least one of IL-2/IL-4/IL-10/IFN-*γ* cytokines and/or CD40L^+^ when compared to the healthy control subjects. A responder frequency of ≥0.01% was defined as a positive response and, when used as a cut-off, it had 100% sensitivity and 88% specificity in discriminating the two groups. The specificity of this test was increased to 100% if only the IFN-*γ* and CD40L^+^ results were taken into consideration but at a corresponding loss of sensitivity. Of note, PDD-induced responses failed to reach significant differences between the TB and non-TB groups and the same cut-off for PDD-specific immune response of responder had 100% sensitivity but 0% specificity [18].

Table 2 summarizes the flow cytometry-determined biomarkers for distinguishing active TB from non-active states.

## 6. Flow Cytometry in the Setting of Immunosuppression

Flow cytometry seems to be advantageous over IGRAs and, especially, TST in the setting of immunosuppression. In the only aforementioned study including HIV patients (indicative of the lack of relevant studies in this population), the performance of the flow cytometry was unaffected by HIV status [37]. Two proof-of-concept studies compared the agreement of flow cytometry assay detecting CD25/CD134 coexpression (a phenotype potentially corresponding to *Mtb*-specific Treg cells and Th17 responses) with QFT-GIT and TST in the detection of recall immune response to TB. The CD25/CD134 assay, QFT-GIT, and TST were performed on 74 participants referred for TB screening in Sydney and on 50 participants with advanced HIV infection (CD4 ≤ 350 × 10^6^ cells/L) in Bangkok. The agreement between CD25/CD134 assay and QFT-GIT was 93.2% in Sydney and 90% in Bangkok. Discordant results occurred around the cut-off of both tests. The agreement between CD25/CD134 assay and TST was 73.6% in Sydney and 84% in Bangkok. The authors concluded that flow cytometry showed good agreement with QFT-GIT in detecting recall response to TB both in well- and less resourced settings in persons with advanced HIV infection and it has the potential to become a useful test for the diagnosis of latent TB, particularly in the setting of HIV infection [38].

In a study by Sester et al. [6] a quantitative flow cytometric whole-blood assay and the TST were comparatively evaluated towards both diagnostic power and practicability in 117 renal transplant recipients on long-term immunosuppressive maintenance therapy [6]. Despite immunosuppression, prevalence (52.14%) and median frequencies of PPD-specific T-cells detected by flow cytometry were as high as previously reported for immunocompetent individuals and haemodialysis patients confirming that the test result is not adversely affected by immunosuppression. In contrast, TST reactivity was significantly reduced as only 50% of patients with PPD reactivity in vitro were TST positive [6].

## 7. Comparison of Flow Cytometry with IGRAs

As both IGRAs and flow cytometry rely on the same principle of inducing INF-*γ* after stimulation with specific antigens, it is not surprising that a significant correlation between T-cell responses detected by flow cytometry and the results obtained by IGRAs has been reported. Studies comparing the two methods showed equivalent results on recall response to TB [38], the PDD-specific [31] and the* Mtb-*specific IFN-*γ* response [35], and the frequencies of PDD-specific dual IFN-*γ*/IL-2-secreting CD4^+^ T-cells [12] as measured by the two methods in various populations and stages of mycobacterial infection. Likewise, a positive correlation was found between the amount of *Mtb*-specific IFN-*γ* measured by QFT-GIT and the frequency of CD69^+^IFN-*γ* ^+^ CD4^+^ T-cells (a phenotype correlated with *Mtb* antigen burden in the same study) measured with flow cytometry [22]. In contrast, a very interesting finding reported by Harari et al. is that *Mtb*-specific IFN-*γ* response measured by ELISpot assay is not significantly different between the two states of infection whereas flow cytometry-measured TNF-*α* clearly distinguishes between active and latent TB [25]. The latter suggests that simultaneous measurement of antigen-specific T-cells cytokines potentially improves the diagnostic capacity.

From a technical point of view, drawbacks of flow cytometry are the fact that it currently compares unfavorably with IGRAs with regard to standardization, interuser, and interlab variability whereas data acquisition/analysis is less easy to perform, more time consuming, and more expensive than IGRAs. Finally, since detection by flow cytometry occurs prior to cytokine release, there is the potential for misleading results due to posttranslational modulation of protein expression [34]. Nonetheless, flow cytometry can be applied within the standard clinical laboratory setting and if performed to a high level, that includes careful titration of fluorescent antibodies, dead cell discrimination, and use of a dump channel to exclude B cells and monocytes, the sensitivity can be greatly improved [39].

## 8. Comparison of Flow Cytometry with TST

A study conducted in Austria by Nemeth et al. assessed the diagnostic value of flow cytometry using TST as gold standard test and showed excellent concordance between the two tests [40]. This small study of 52 BCG-vaccinated patients with dermatological disorders, tested prior to implementation of anti-TNF-*α* therapy, measured the expression of *Mtb*-specific IFN-*γ*, TNF-*α*, IL-2, and IL-10 in CD4^+^ and CD8^+^ T-cells. Among all the phenotypes, *Mtb*-specific IFN-*γ* ^+^ CD4^+^ T-cells showed a good correlation with TST results. The highest concordance with the TST was shown for CD4^+^ T-cells coexpressing TNF-*α* and IFN-*γ*. CD4^+^ T-cells, expressing both IL-2 and IFN-*γ*, displayed high specificity as well, suggesting that measurement of cytokine coproducing T-cells may, overall, be more specific for LTBI than measurement of only one cytokine. Noteworthy, the high degree of agreement between the two methods should be interpreted in the context of low exposure to NTM in Central Europe and the practice of administration of BCG vaccine in early childhood in the study setting thus minimizing the effect of BCG vaccination on TST. Putting it all together, the enumeration of *Mtb*-specific CD4^+^ T-cells might actually introduce greater specificity for the diagnosis of latent TB, compared to the TST [40].

In another study evaluating 97 patients with inflammatory arthropathies before treatment with TNF-*α* blocking drugs, TST as a part of a diagnostic algorithm also including history and chest X-ray had a low sensitivity and specificity for the diagnosis of LTBI, potentially resulting in both over- and undertreatment with prophylactic INH when compared with the flow-cytometric analysis of whole-blood T-cell reactivity to proteins specific to *Mtb* [7].

## 9. Conclusion

Flow cytometry analysis holds promise for becoming a novel method that will assist the decrypting of the specific immune profile associated with different stages of infection, the enhanced classification of subjects with *Mtb* infection, either active, latent, recently acquired, or successfully treated in routine clinical practice, the development of immune-based interventions, and ultimately the control of *Mtb*. Of the main advantages of flow cytometry compared to IGRAs is the capacity to simultaneously measure more than one of the several cytokines secreted by antigen-specific T-cells thus potentially improving the diagnostic capacity. Additionally, flow cytometry is not hindered by the presence of immunosuppression, is not confounded by BCG vaccination, and is less frequently cross-reacting with NTM. Nonetheless, it is essential that flow cytometry becomes easier to be performed and analyzed before its advantages can be converted into wider clinical benefit. Finally, in order to confirm flow cytometry's broader utility and applicability, larger and metacentric studies involving patients with HIV infection and/or other forms of immunosuppression as well as patients with multidrug-resistant TB, TB suspects with a lung disease other than tuberculosis, or patients in the setting of contact tracing are indispensable.

## Figures and Tables

**Table 1:** Comparison of tuberculin skin test (TST), interferon gamma release assay (IGRA), and flow cytometry assay.

	TST	IGRAs	Flow Cytometry
Method	A valid TST requires proper administration by the Mantoux method with intradermal injection of 0.1 mL of tuberculin PPD into the volar surface of the forearm	A single specimen of peripheral blood is drawn and incubated in vitro overnight with *Mtb*-specific antigens	IntacT-cells and their constituent components are tagged with fluorescently conjugated monoclonal antibodies and/or stained with fluorescent reagents and then analyzed individually. Cells and the fluorescent molecules in/on each cell are excited by passing through the laser light at speeds exceeding 70,000 cells per second. Each cell passing through the beam also scatters light providing an indication of cell shape and size

Characteristics	A delayed-type hypersensitivity to intradermal injection of tuberculin-PPD testing for cell-mediated immunity against *Mtb *	The QFT-GIT measures the amount of INF-*γ* whereas the T-Spot determines the number of cells producing INF-*γ*; therefore they provide limited information regarding the complete phenotype of cells engaged in cytokine production or the kinetics of this response [19]	Allows analysis of multiple parameters per cell and accurately locates the pool of immunological effector cells responsible for cytokine production [19]

Sensitivity (%)	0.65	QFT-G-IT 0.80 T-SPOT.*TB *0.81 [8]	See Table 2

Specificity (%)	0.75	QFT-G-IT 0.79 T-SPOT.*TB * 0.59 [8]	See Table 2

T-cell populations detected	Primary central memory T-cells [6]	Primary effector memory T-cells [6]	All T-cell populations can be detected

TB stage	More likely to identify persons with longstanding cellular immune responses to these antigens [6]	More likely to identify persons who have recently been infected with *M. tuberculosis,* a group at particularly high risk for progression to disease [6]	Promising tool for the identification of all stages of TB infection

Immunosuppression	Compromised performance [9, 33]	Compromised performance [9, 10]	Unaffected performance [31–33]

Cross-reactivity with BCG	Yes [3, 36]	No	No [31]

Cross reactivity with NTM	Yes (in 18 studies involving 1,169,105 subjects, the absolute prevalence of false-positive TST from NTM cross-reactivity ranged from 0.1% to 2.3% in different regions [36])	Yes, but less extensive than TST (ESAT-6 and CFP-10 are present in MTM *M. kansasii, M. szulgai, *and *M. marinum*, and sensitization to these organisms might cause false-positive IGRA results [3])	No (evidence suggests that flow cytometry might actually discriminate between infection with *Mtb* or NTM [37])

“Booster” phenomenon	Yes	No	No

Patient visits	2	1	1

Processing time		Within 16 (QFT-GIT) to 30 (T-spot) hours	45 minutes–1 hour (average)

Time to results	48–72 hours	Within 16 (QFT-GIT) to 30 (T-spot) hours	Within 24 hours (~8 h) [35]

Interreader variability	Yes	No (however, careful interpretation of true rather than artifactual (nonspecific) reactions is essential when the number of spots is counted in T-spot)	Expertise is required for correct and reproducible gating

Settings	Errors in intradermal administration, interpretation, and interreader variability	Errors in collecting or transporting blood specimens or in running and interpreting the assay can decrease the accuracy of IGRAs	Due to the need for technical expertise and expensive equipment, it is recommended that this assay be done only in a reference laboratory setting

NTM: nontuberculous mycobacteria; BCG: Bacille de Calmette et Guérin.

**Table 2:** Flow cytometry: determined biomarkers for distinguishing active TB from nonactive states.

Reference	Population studied	Population validated	Biomarker	Sensitivity (%)	Specificity (%)	PPV (%)	NPV (%)
[30]	36	31	% PPD-reactive CD27^−^IFN-*γ* ^+^ CD4^+^ T-cells > 48	100	100	100	100

[19]	40	—	% ESAT-6/CFP-10-reactive IFN-*γ* ^+^ CD4^+^ T-cells > 0.01	94.1	36.4		

[4]	52	50	% PPD-reactive IFN-*γ* ^+^IL-2^+^ CD4^+^ T-cells < 56	70	100		

[31]	42	—	% PPD-specific IFN-*γ* ^+^ CD4^+^ T-cells ≥ 0.5	89	80		

[21]	48	101	% ESAT-6/CFP-10-reactive single-positive TNF-*α* CD4^+^ T-cells > 37.4	67	92	80	92.4

[16]	31	—	% ESAT-6-reactive CD69^+^ CD4^+^ T-cells producing at least one of the following cytokines: IL-2/IL-4/IL-10/IFN-*γ* and/or CD40L^+^ ≥ 0.01	100	88		

PPV: positive predictive value; NPV: negative predictive value.
